# Age-related hearing loss in the Korea National Health and Nutrition Examination Survey

**DOI:** 10.1371/journal.pone.0243001

**Published:** 2020-12-01

**Authors:** Subin Kim, Jung Mee Park, Jae Sang Han, Jae Hyun Seo, Kyung-Do Han, Young Hoon Joo, Kyoung Ho Park

**Affiliations:** 1 Department of Otorhinolaryngology, College of Medicine, The Catholic University of Korea, Seoul, Republic of Korea; 2 Department of Statistics and Actuarial Science, Soongsil University, Seoul, Republic of Korea; Ehime University Graduate School of Medicine, JAPAN

## Abstract

**Objectives:**

Age-related hearing loss (ARHL), also known as presbycusis, is a chronic disorder characterized by impairment of the transduction of acoustic signals. This study analysed the prevalence and demographic characteristics of ARHL in the Korean population.

**Methods:**

We used the data from the Korea National Health and Nutrition Examination Survey (KNHANES) from 2009 to 2012 and analysed the association between age and hearing impairment. A total of 16,799 adults were selected for the current study. Physical examinations, blood tests, otoscopic examinations, and hearing tests were performed. The demographic variables included age, gender, obesity, economic status, education level, noise exposure history, and underlying diseases.

**Results:**

Among 16,799 participants, the prevalence of unilateral hearing loss was 8% (1,349 people), and bilateral hearing loss was 5.9% (989 people). Men were 53.4% more likely to have hearing loss than women. Age and underlying diseases, like hypertension, diabetes, and abdominal obesity, were significantly associated with hearing loss (P < 0.0001). Further, mental health factors, such as cognitive function, depression, and suicidal ideation, were related to hearing loss. The prevalence of hearing loss increased with advancing years, especially in the high frequency of 6 kHz, with a sharply increase in patients aged 65 and over.

**Conclusion:**

The analysis of auditory performance in the Korean population confirmed the association of high-frequency hearing loss with advancing age. A threshold of 6 kHz should be included to correctly diagnose hearing impairment in elderly patients. Patients with ARHL should be provided with suitable aural rehabilitation that includes active high-frequency control.

## Introduction

Age-related hearing loss (ARHL), also known as presbycusis, is a chronic disorder characterized by impairment of the transduction of acoustic signals. As the auditory organ ages, poor sound discrimination occurs due to increases in the hearing threshold and decreases in central nervous system processing [1]. Multiple factors, such as a loss of hair cells in the cochlea, metabolic disturbances by dysfunction of the stria vascularis, and degeneration of the spiral ganglion cells associated with aging, lead to difficulty in cochlear amplification.

ARHL especially misses higher-pitched consonant sounds like /s/, /t/ or /f/ and usually shows symmetrical sensory neural hearing loss of high frequencies, which affects understanding in noise. Difficulty hearing in noise is one of the first signs of sensory neural hearing loss [2]. The chief patient complaint is usually difficulty understanding during conversations rather than hearing impairment itself and leads to low discrimination scores in the speech-in-noise test. When it progresses to 2–4 kHz, it affects speech understanding in most situations [3].

In the United States, hearing impairment, which is experienced by 13% of the total population, was reported to be age-related [4]. According to the Korean Ministry of Health and Welfare statistics annual report for 2000, hearing impairment was experienced by 7.2% of the population aged 65 in 2000 and was forecasted to reach 13.2% by 2020. ARHL causes word confusion and conversational breakdown, social isolation, and affects an individual’s psychosocial status. Moreover, hearing impairment is known to be related to cognitive status in patients with dementia [5]. In this respect, ARHL is becoming an emerging national health issue. Early diagnosis, prevention, and rehabilitation should be undertaken to improve the welfare and quality of life for the elderly.

This study analysed the prevalence and demographic characteristics of ARHL in the Korean population using data from the Korea National Health and Nutrition Examination Survey (KNHANES) 2009–2012.

## Materials and methods

### Study design and subjects

The KNHANES data, which is a national surveillance system that has been providing the health and nutritional status of Koreans since 1998 was analysed retrospectively. Based on the National Health Promotion Act, the surveys have been conducted by the Korea Centers for Disease Control and Prevention (KCDC) [6]. The survey includes a health interview, a nutritional survey, and a health examination survey via household interviews or direct physical examinations by professions. The KNHANES data and research methodology is officially guided in KNHANES website (https://knhanes.cdc.go.kr/knhanes/eng/index.do). The KNHANES annual reports from 1998 to currently 2018 are available in the website, and raw data are also available on request.

KNHANES data from 2009 to 2012 were used for the current study to estimate the association between age and hearing impairment. A total of 16,799 adults were selected, and those under 19 years old were excluded. Physical examination data, such as body mass index (BMI), and blood, otoscopic, and hearing test (pure tone audiometry at 500 Hz, 1000 Hz, 2000 Hz, 3000 Hz, 4000 Hz, and 6000 Hz) results were collected. The demographic variables included age, gender, obesity, income, education level, noise exposure, and known underlying diseases, such as hypertension, diabetes mellitus, and depressive disorders. Audiometric social hearing loss was defined as average pure tone thresholds of greater than 25 dB in the better-hearing ear at 0.5, 1, 2, and 4 kHz.

### Data analysis

Statistical analyses were performed using SAS version 9.4 (SAS Institute, Cary, North Carolina, USA). T-test was used for continuous variables and chi-square test was used for categorical variables. A p-value less than 0.0001 was deemed to indicate statistical significance.

### Ethics statement

The data of KHANES was obtained with the written informed consent from all participants prior to the survey. Based on the National Health Promotion Act, the surveys have been conducted by the Korea Centers for Disease Control and Prevention (KCDC), and anonymised raw data are provided to the researchers under request. This study was carried out in accordance with the Declaration of Helsinki on biomedical research for human subjects and was approved by the Institutional Review Board of Seoul St. Mary’s hospital (approval no. KC12EIME0077).

## Results

### Demographic data

Among 16,799 participants, the prevalence of unilateral hearing loss was 8.2% (1,381), and bilateral hearing loss was 5.3% (883). Mild hearing impairment (26–40 dB) was present in 51.7% (784) of the participants with unilateral hearing loss. Moderate hearing impairment (41–70 dB) was present in 86.8% (762) of the participants, whereas severe hearing impairment (> 70 dB) was present in 13.2% (121) of the participants with bilateral hearing loss.

The characteristics of the study participants were shown in Table 1. Men were 53.4% more likely to have hearing loss than women. Mental health factors, such as depression and suicidal ideation, were also related to hearing impairment. Hearing impairment was significantly associated with age, diabetes mellitus, hypertension, abdominal obesity, level of education, income, living area, tinnitus, and dizziness (*P* < 0.0001).

**Table 1:** Characteristics of the study participants (N = 16,799).

Characteristics	Hearing Impairment		*P*-values
	Yes (%)	No (%)	
**Sex (Male)**	53.4(1.7)	49.3(0.4)	0.0254
**Age**	58.9±0.8	44.3±0.2	**< 0.001**
**Marriage (Spouse)**	76.4(1.5)	80.7(0.7)	0.0044
**Diabetes mellitus**	15.3(1.4)	7.9(0.3)	**< 0.001**
**Hypertension**	46.8(1.9)	25.6(0.5)	**< 0.001**
**Systemic Obesity**	33.2(1.7)	32.1(0.5)	0.5243
**Abdominal Obesity**	40.3(1.8)	32.1(0.6)	**< 0.001**
**Education**	43.6(2.3)	73.7(0.6)	**< 0.001**
**Income (Low 30%)**	31(1.7)	14.7(0.5)	**< 0.001**
**Place (Rural)**	29.6(2.8)	19(1.6)	**< 0.001**
**Smoking**	23.2(1.7)	23.9(0.5)	0.6575
**Alcohol**	9.2(1.2)	10.1(0.3)	0.4574
**Exercise**	16.8(1.4)	20(0.5)	0.0342
**Noise exposure**	26.6(1.8)	24.1(0.9)	0.1245
**Tinnitus**	37.9(1.8)	20.2(0.5)	**< 0.001**
**Dizziness**	15.5(1.4)	7.5(0.3)	**< 0.001**
**Stress**	25.8(1.6)	27.8(0.5)	0.2431
**Depression**	17(1.3)	12.7(0.3)	0.0002
**Suicidal ideation**	20(1.4)	13.4(0.4)	**< 0.001**
**Falls**	3.1(0.6)	1.3(0.1)	**< 0.001**

Values are presented as number (%). T-test was used for continuous variables and chi-square test was used for categorical variables.

### Age-related high-frequency hearing impairment

**Fig 1:**
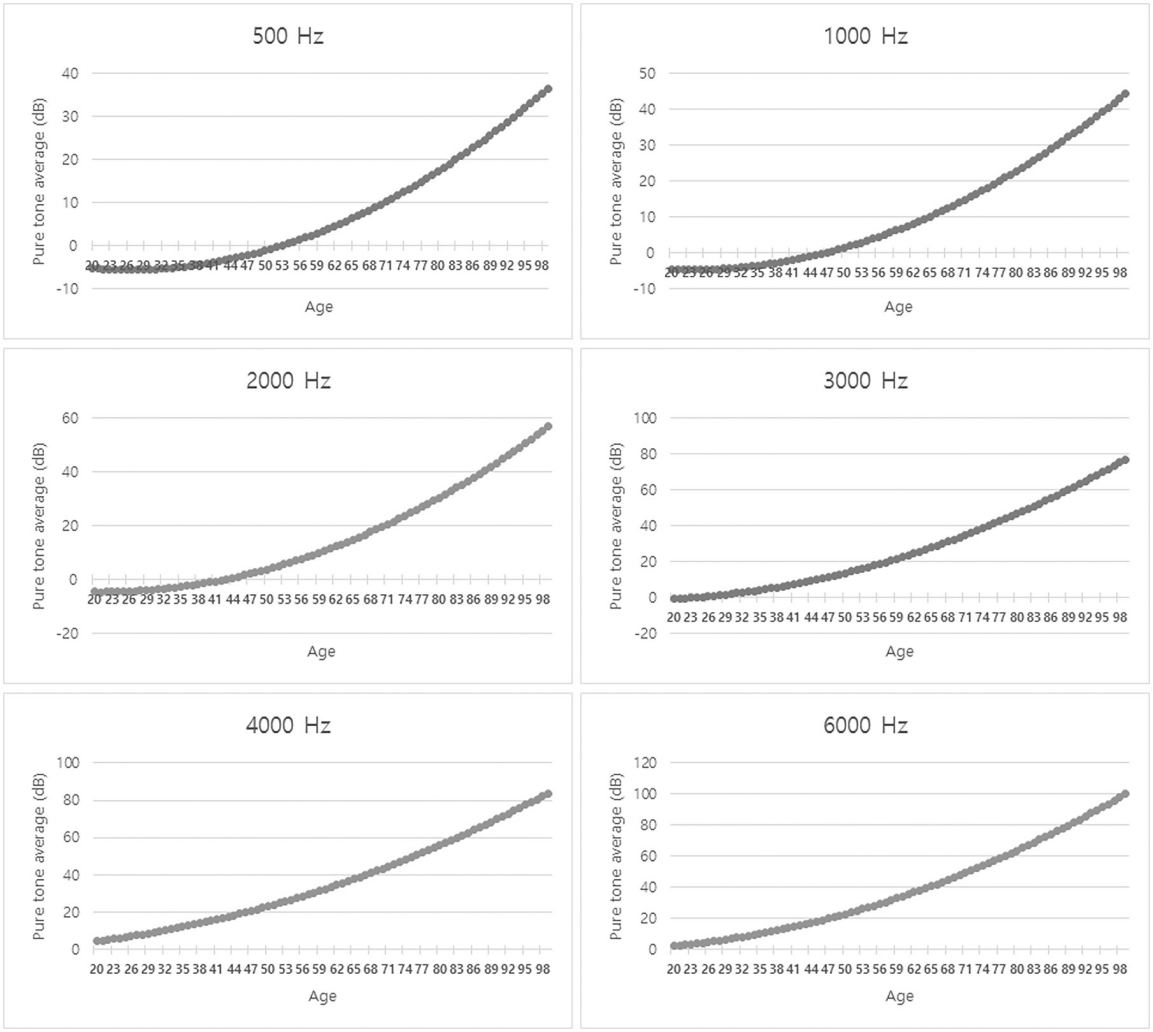
Pure tone average in the better ear at each age (500 Hz, 1000 Hz, 2000 Hz, 3000 Hz, 4000 Hz, and 6000 Hz). The average value was increased with age at all frequencies, especially at high frequencies.

**Fig 2:**
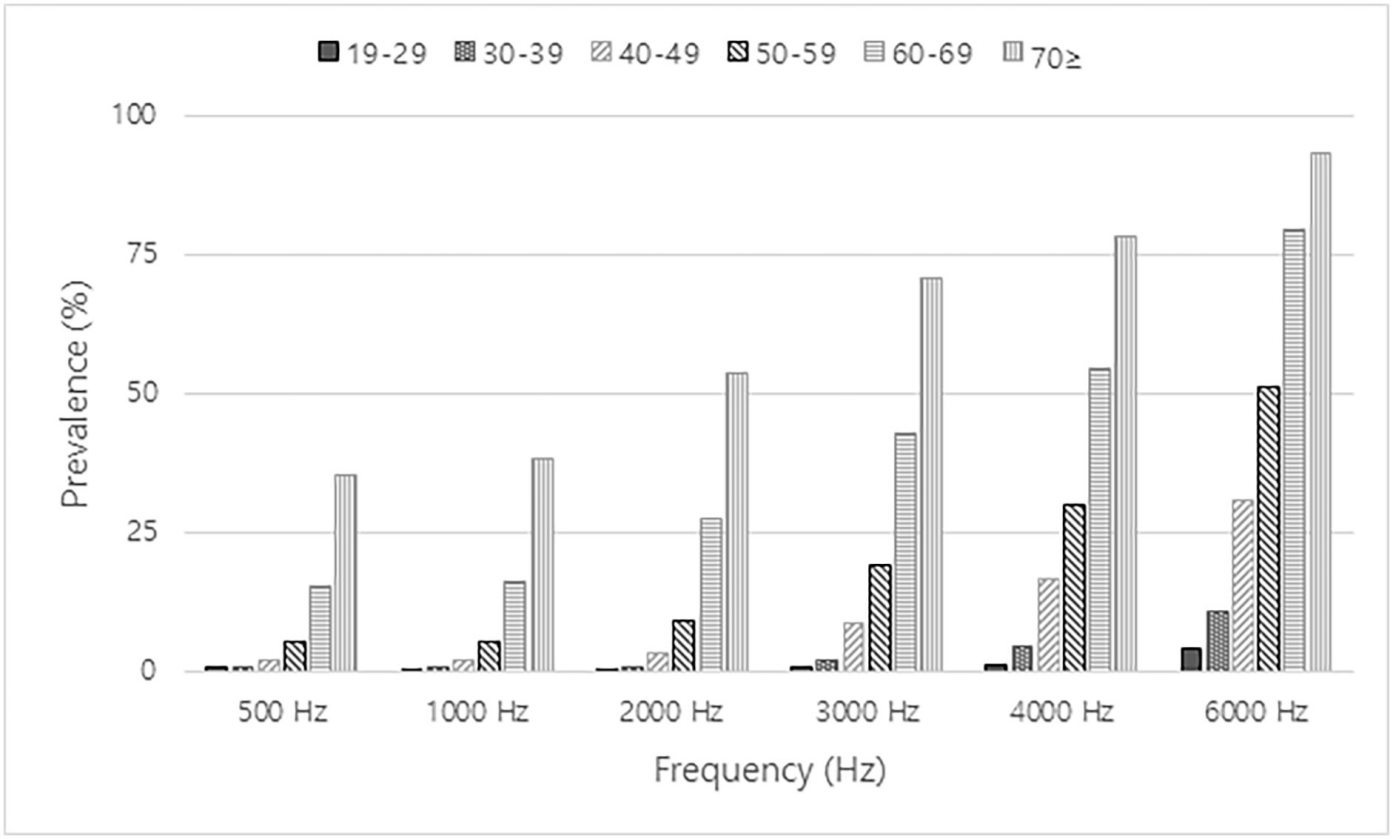
Prevalence of hearing impairment (more than 25 dB in the better ear) at each frequency by decade age (2009–2012). The prevalence was steeply increased with age at all frequencies.

The pure tone average in the better ear of the participants of each age showed a curved to linear graph over all frequencies (500 Hz, 1000 Hz, 2000 Hz, 3000 Hz, 4000 Hz, and 6000 Hz) (Fig 1). As shown in Table 2, the prevalence of hearing impairment was statistically related to the age (P < 0.0001). This result is graphically shown in Fig 2. The prevalence of hearing impairment increased with advancing years at each frequency. The prevalence of moderate hearing impairment, which was defined as the average of pure tone audiometry at 0.5, 1, 2 and 4k Hz more than 40 dB in the better ear, also steeply increased with age, regardless of gender.

**Table 2:** Prevalence of hearing impairment (more than 25 dB in the better ear) at each frequency by decade age.

		Prevalence (%)					
	Age	500 Hz	1000 Hz	2000 Hz	3000 Hz	4000 Hz	6000 Hz
Total	19–29	0.7(0.2)	0.4(0.2)	0.4(0.2)	0.8(0.2)	1.3(0.3)	4(0.5)
	30–39	0.9(0.2)	0.6(0.2)	0.9(0.2)	1.9(0.3)	4.3(0.4)	10.7(0.7)
	40–49	1.9(0.3)	1.8(0.3)	3.1(0.4)	8.7(0.6)	16.5(0.7)	30.8(0.9)
	50–59	5.2(0.4)	5.4(0.5)	9.2(0.6)	18.9(0.9)	29.9(0.9)	51.3(1.1)
	60–69	15.2(0.9)	16(0.8)	27.4(1)	42.7(1.1)	54.3(1.1)	79.5(0.9)
	70≥	35.3(1.2)	38.2(1.3)	53.7(1.2)	70.7(1.1)	78.4(1)	93.2(0.6)
	P-value	< .0001	< .0001	< .0001	< .0001	< .0001	< .0001
Male	19–29	0.9(0.4)	0.5(0.3)	0.4(0.2)	1(0.4)	1.5(0.4)	4.8(0.9)
	30–39	0.8(0.3)	0.5(0.2)	1.1(0.3)	2.9(0.5)	7.2(0.8)	14.9(1)
	40–49	1.7(0.4)	2(0.4)	3.9(0.6)	14.1(1)	28.5(1.3)	39.8(1.4)
	50–59	4.7(0.6)	5(0.7)	10.4(1)	27.4(1.5)	45(1.6)	59.8(1.6)
	60–69	13.7(1.2)	15(1.2)	29.5(1.6)	51.7(1.6)	69.8(1.4)	85.2(1.1)
	70≥	31.5(1.6)	35.3(1.8)	54.2(1.7)	75.3(1.5)	86.6(1.3)	94.7(0.8)
	P-value	< .0001	< .0001	< .0001	< .0001	< .0001	< .0001
Female	19–29	0.4(0.2)	0.3(0.2)	0.5(0.3)	0.6(0.3)	1(0.4)	3(0.6)
	30–39	1(0.3)	0.8(0.3)	0.6(0.2)	0.8(0.2)	1.2(0.3)	6.3(0.7)
	40–49	2.2(0.4)	1.6(0.3)	2.4(0.4)	3(0.5)	4(0.5)	21.4(1.1)
	50–59	5.6(0.6)	5.7(0.7)	8.1(0.7)	10.7(0.8)	15.1(1)	42.9(1.4)
	60–69	16.6(1.2)	16.9(1.2)	25.3(1.3)	34.2(1.4)	39.7(1.5)	74(1.4)
	70≥	37.9(1.6)	40.3(1.7)	53.4(1.6)	67.5(1.6)	72.7(1.4)	92.2(0.8)
	P-value	< .0001	< .0001	< .0001	< .0001	< .0001	< .0001

The prevalence of hearing impairment increased with advancing years in all frequencies. Values are presented as number (%) and Chi-square test was used to analyse the statistical difference between the prevalence rates of each group.

## Discussion

Hearing loss is known to be highly dominant among people older than 70 years [7]. Based on data from the National Health and Nutrition Examination Survey (NHANES) of the United States, mild severity (pure tone average thresholds of >25 dB) bilateral hearing loss doubled for every 10 years of age after 50 [8, 9]; 15% of people between the ages of 50 and 59, 31% of those between the ages of 60 and 69, and 63.1% of those aged 70 years and older. Among people aged 85, the prevalence was 80% [8]. Similarly, hearing loss in our study showed a steeply increasing curve starting at age 60 at every frequency (Fig 1).

**Fig 3:**
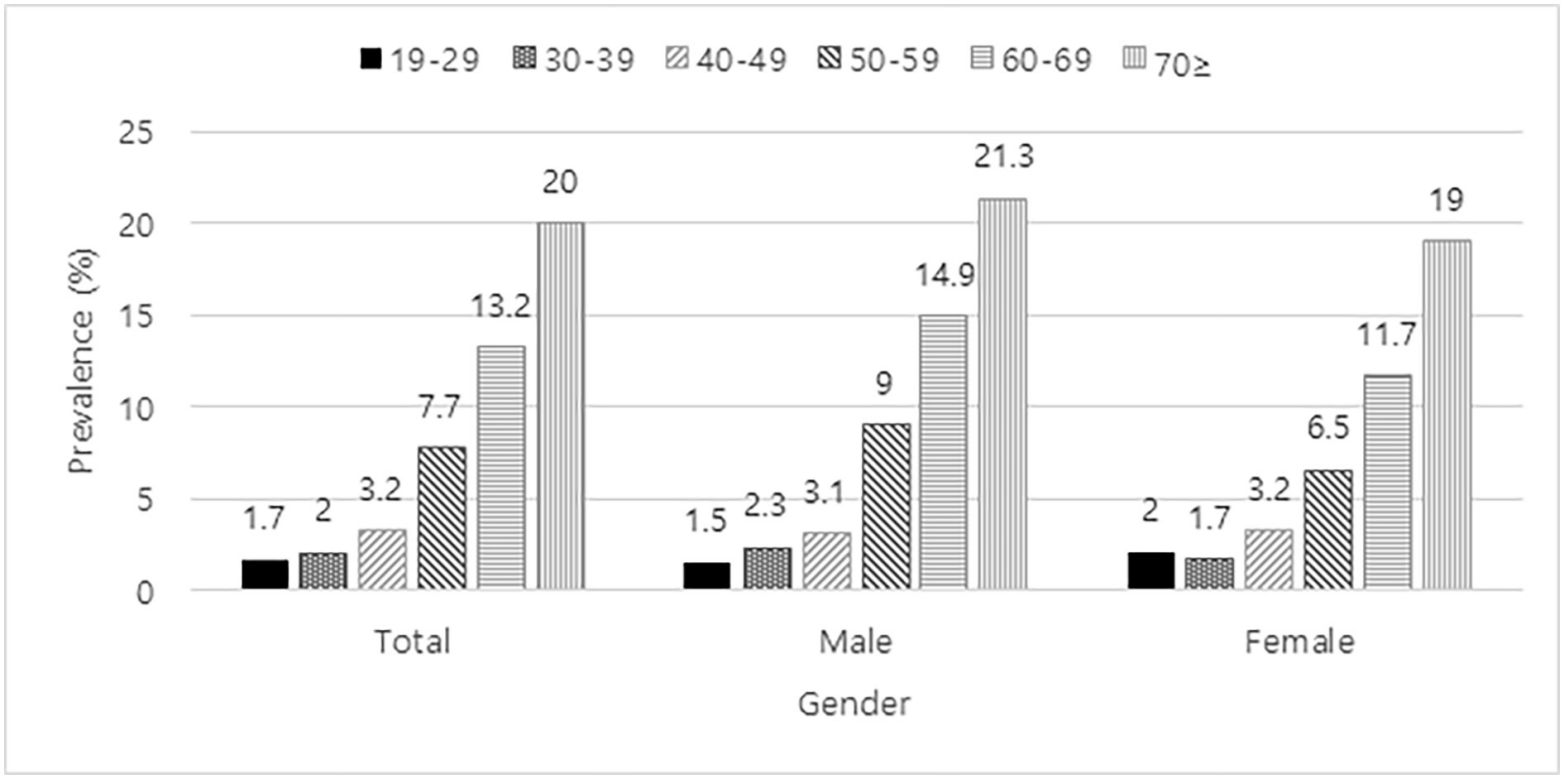
Prevalence of moderate hearing impairment (average of pure tone audiometry at 0.5, 1, 2 and 4k Hz more than 40 dB in the better ear). An increasing prevalence over advancing years was observed regardless of gender.

In Korea, the statistical annual report from the Ministry of Health and Welfare in 2000 reported that 7.2% of people over age 65 had a hearing impairment. This report also predicted the prevalence to be 13.2% in 2020. However, our study, which was based on the KNHANES 2009–2012 data, showed that the prevalence of hearing impairment had already surpassed the expectation. As shown in Fig 3, the prevalence in patients between the ages of 60 and 69 was already 13.2%. For people in their 70s, the prevalence rate was 20%. This indicates that age-related hearing loss is progressing faster than expected, making it an important national health priority.

The demographic data of the KNHANES showed several characteristics related to hearing impairment (Table 1). Diabetes mellitus and hypertension showed statistically high relationships with hearing loss (*P* < 0.0001). Other previous reports also suggested that the duration and severity of diabetes were possible significant predictors of hearing impairment. Bilateral sensory neural hearing loss was a complication associated with type 2 diabetes mellitus [10]. Systolic blood pressure showed a significant relationship with hearing loss in several reports [11]. Since hypertension and diabetes mellitus are relatively modifiable risk factors, this suggests that preventing and managing these diseases might help to effectively prevent hearing loss.

Several studies have shown that smoking increased the prevalence of hearing loss [12–17]. Unexpectedly, smoking was not associated with hearing loss in our data analysis. Data on alcohol consumption showed diverse results in many reports. In some studies, hearing loss was associated with chronic alcohol abuse and related to the period and amount of abuse history [15]. However, moderate alcohol intake was not significantly related to hearing loss [11] or even had a protective effect in another study [7]. In our data, alcohol consumption had no significant effect on hearing loss. People with the above risks are recommended to undergo frequent screening for hearing impairment.

This study has a limitation in that the data did not reflect the latest 5 year survey result. The Database of Korea National Health and Nutrition Examination Survey (KNHANES) has been performed since 1998. However, hearing test was first included as a health examination component since 2008. We excluded the data of the first year, and the data of next three years were analyzed (2009–2012). Currently, the raw data of 2018 was released in May, 2020. In future study, it will be better to include the latest survey result from 2013 to 2018 to analyze a decade survey of Korean hearing test (2009~2018).

Hearing impairment negatively impacts the quality of life, especially the social and emotional aspects of communication. However, people, especially those of old ages, often think of it as an inevitable aging phenomenon and do not readily undergo medical examinations. According to the National Center for Health Statistics report in 2010, only 41% of US adults aged 70 years and older reported having had a hearing examination in the past five years [9]. Since the prevalence of hearing impairment steeply increases with advancing years, the early diagnosis of age-related hearing loss is crucial. The accessibility of screening tests needs to be high for the elderly population to achieve early hearing rehabilitation and better quality of life.

## Conclusion

Age-related hearing loss (ARHL) is a major disease that affects the quality of life of elderly people. The analysis of the auditory performance in the Korean population indicated the importance of high-frequency hearing loss in the elderly. People who are with risk for hearing loss should undergo frequent screening for hearing impairment. We also emphasize the necessity of early proper aural rehabilitation that includes hearing aids, with active frequency control for elderly patients with hearing impairment.
